# Molecular Modeling Study for the Design of Novel Peroxisome Proliferator-Activated Receptor Gamma Agonists Using 3D-QSAR and Molecular Docking

**DOI:** 10.3390/ijms19020630

**Published:** 2018-02-23

**Authors:** Yaning Jian, Yuyu He, Jingjing Yang, Wei Han, Xifeng Zhai, Ye Zhao, Yang Li

**Affiliations:** 1 Biomedicine Key Laboratory of Shaanxi Province, The College of Life Sciences, Northwest University, Xi’an 710069, China; yaningjian001@163.com (Y.J.); heyuyu_66123@163.com (Y.H.); yangjing952017@163.com (J.Y.); 18629670486@163.com (W.H.); zhaoye@nwu.edu.cn (Y.Z.); 2 Key Laboratory of Resource Biology and Biotechnology in Western China, Ministry of Education, Northwest University, Xi’an 710069, China; 3 School of Pharmaceutical Sciences, Xi’an Medical University, Xi’an 710021, China; zhaixf@xiyi.edu.cn

**Keywords:** PPARγ, *N*-benzylbenzamide derivatives, 3D-QSAR, CoMFA, CoMSIA, molecular docking

## Abstract

Type 2 diabetes is becoming a global pandemic disease. As an important target for the generation and development of diabetes mellitus, peroxisome proliferator-activated receptor γ (PPARγ) has been widely studied. PPARγ agonists have been designed as potential anti-diabetic agents. The advanced development of PPARγ agonists represents a valuable research tool for diabetes therapy. To explore the structural requirements of PPARγ agonists, three-dimensional quantitative structure–activity relationship (3D-QSAR) and molecular docking studies were performed on a series of *N*-benzylbenzamide derivatives employing comparative molecular field analysis (CoMFA), comparative molecular similarity indices analysis (CoMSIA), and surflex-dock techniques. The generated models of CoMFA and CoMSIA exhibited a high cross-validation coefficient (*q*^2^) of 0.75 and 0.551, and a non-cross-validation coefficient (*r*^2^) of 0.958 and 0.912, respectively. The predictive ability of the models was validated using external validation with predictive factor (*r*^2^_pred_) of 0.722 and 0.682, respectively. These results indicate that the model has high statistical reliability and good predictive power. The probable binding modes of the best active compounds with PPARγ active site were analyzed, and the residues His323, Tyr473, Ser289 and Ser342 were found to have hydrogen bond interactions. Based on the analysis of molecular docking results, and the 3D contour maps generated from CoMFA and CoMSIA models, the key structural features of PPARγ agonists responsible for biological activity could be determined, and several new molecules, with potentially higher predicted activity, were designed thereafter. This work may provide valuable information in further optimization of *N*-benzylbenzamide derivatives as PPARγ agonists.

## 1. Introduction

Type 2 diabetes (T2D) is a disease that is generally characterized by relative insulin deficiency caused by insulin resistance in target organs, and pancreatic β-cell dysfunction [1]. In 2014, there were 422-million people with diabetes, with more than 90% estimated to have T2D, worldwide. Unfortunately, this number will increase to approximately 552-million by the year 2030 [2]. Accordingly, T2D is generating a significant socioeconomic burden, as a pandemic disease with a high and increasing fatality [3,4].

The peroxisome proliferator-activated receptor γ (PPARγ) is generally regarded as a molecular target for the thiazolidinedione class of anti-diabetic drugs [5,6], as it plays a key role in the generation and development of diabetes mellitus [7,8,9]. Recent studies have shown that PPARγ agonists, including rosiglitazone and pioglitazone [10], may be used as insulin sensitizers in target tissues to lower glucose, as well as fatty acid levels in T2D patients.

However, both rosiglitazone and pioglitazone have been withdrawn from the market because of significant hepatotoxicity and cancer development concerns [11]. Hence, there is an urgent need for the development of safer PPARγ modulating drugs. One severe side-effect of known PPARγ agonists, involves sodium and water retention, which may be dangerous for patients suffering from congestive heart conditions [12]. Recently, various new *N*-benzylbenzamide compounds have been shown to act as PPARγ agonists that, not only lowered blood pressure and reduced systemic glucose, triglycerides, and free fatty acid levels, but have also been shown to maintain water and electrolyte homeostasis [13]. Therefore, a variety of *N*-benzylbenzamide compounds have since been identified as safer PPARγ modulators for the treatment of T2D.

Based on CoMFA [14], along with CoMSIA [15], methods involving 3D-QSAR determinations allow for the structure–activity relationship of *N*-benzylbenzamide compounds to be studied. Molecular docking was also applied to reveal the most likely binding modes between the compounds and PPARγ. On the basis of 3D-QSAR and molecular docking results, valuable information can be retrieved for further structured-based drug design, with higher activity. Finally, a series of new potent molecules with a higher predicted activity than the template compound, the latter exhibiting the best activity reported in the literature, have been designed. Our study will potentially provide guidance for the future design of selective and potent PPARγ agonists.

## 2. Results and Discussion

### 2.1. CoMFA and CoMSIA Results

The 3D-QSAR models were obtained using a training set of 27 compounds, and a test set of six compounds. The statistical parameters associated with CoMFA and CoMSIA can be found in Table 1. In general, various alignment strategies can lead to different statistical values in the constructed QSAR models. The best CoMFA and CoMSIA models were generated employing a partial least square (PLS) analysis, which produced cross-validated coefficients (*q*^2^). When a cross-validation coefficient, *q*^2^ > 0.5, was used, the QSAR model demonstrated statistical significance.

**Figure 1:**
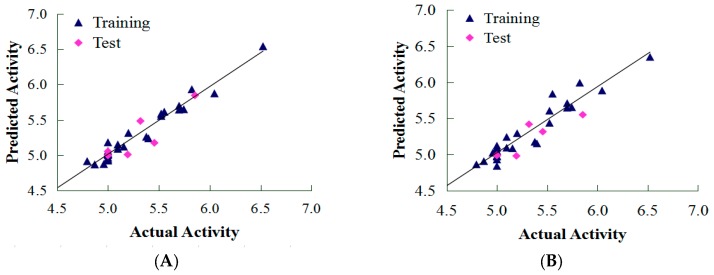
Plots of Actual versus predicted pEC_50_ values, for the training set and test set compounds, for CoMFA (**A**) and CoMSIA (**B**) models.

As shown in Table 1, two descriptor fields in CoMFA form all three possible combination models, including steric (S), electrostatic (E) and SE models. The CoMSIA models, with a combination of five descriptor fields, including S, E, hydrophobic (H), hydrogen bond donor (D) and acceptor (A), were developed to generate the optimal 3D-QSAR model. However, some models with a low *q*^2^ value did not meet the criterion (*q*^2^ > 0.5), indicating an unacceptable 3D-QSAR model. Still, overfitting seemed to occur for some models (those with a large number of components). From Table 1, we can see that the best established models (CoMFA and CoMSIA) exhibited high *q*^2^ (0.75 and 0.551), *r*^2^ (0.958 and 0.912), and F-values (76.113 and 43.388), along with a low standard error of estimate (SEE) value (0.097 and 0.138), and a suitable number of components (6 and 5), which indicated good statistical correlation of the models. Moreover, the predictive capabilities of the generated models were assessed by calculating their predictive correlation coefficient (*r*^2^_pred_) involving their corresponding test set molecules. The generated CoMFA and CoMSIA models with maximum external predictive ability (*r*^2^_pred_ 0.722 and 0.682), were considered the best models. The distribution of actual predicted pEC_50_ values of the training and test sets for CoMFA and CoMSIA are shown in Figure 1. The CoMFA and CoMSIA models show a good fit along the diagonal line. Both models also exhibited satisfactory predictive ability throughout the training and test sets.

### 2.2. CoMFA Contour Map Analysis

**Figure 2:**
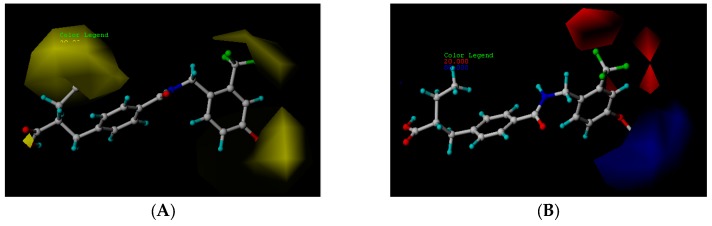
CoMFA contour maps displayed with most potent compound, **24c**. (**A**) CoMFA steric contour map (green, favored; yellow, disfavored); (**B**) CoMFA electrostatic contour map (blue, electropositive favored; red, electronegative favored).

The steric and electrostatic fields of the CoMFA model are presented as contour maps in Figure 2. Finally, compound **24c** was selected as the template molecule. The green contours represent regions indicating favorable steric fields, while the yellow contours represent the regions indicating unfavorable steric fields. Moreover, the blue and red contours highlight the positions where electropositive groups and electronegative groups would be favorable, respectively.

#### 2.2.1. Steric Contour Map

The steric contour map in CoMFA (Figure 2A) has a yellow contour near the ortho position of the benzene ring, which indicates that the presence of steric substituents in this region is unfavorable. Furthermore, the yellow contour explains why a –CF_3_ substituent in ortho position of the benzene ring in compound **2b** is more potent than in compound **10b**, which bears a –OCF_3_ substituent. Likewise, a small yellow contour map appeared in the para position of the benzene ring, which indicates that the large size of the substituent was not preferred in this area. Moreover, a –OCH_3_ group in this position in compound **18b**, could be found within the steric field, which led to decreased biological activity. Finally, the large yellow region on the R_2_ substitutes may explain why compound **32b**, bearing a phenyl group, was less active than compound **30b** bearing a propyl group.

#### 2.2.2. Electrostatic Contour Map

A large blue contour area near the para position of the benzene ring indicates that the presence of an electropositive group may increase activity (Figure 2B). This assumption becomes even more significant in the case of compounds **20b** and **12b**, as these compounds contain a –Cl and –F substituent, respectively. However, due to the presence of different electron-donating groups, compound **12b** was found to be less biologically active than compound **20b**. The red contours present on the ortho position of the benzene ring suggest that an electron negative group would be favorable in this area, an assumption that proves to be true for compounds **2b** and **6b**, which contain a –CF_3_ group and a –CH_3_ group, respectively. However, since a –CF_3_ group proves to be more electron-withdrawing than a –CH_3_ group, compound **2b** was determined to be more biologically active than compound **6b**.

### 2.3. CoMSIA Contour Map Analysis

**Figure 3:**
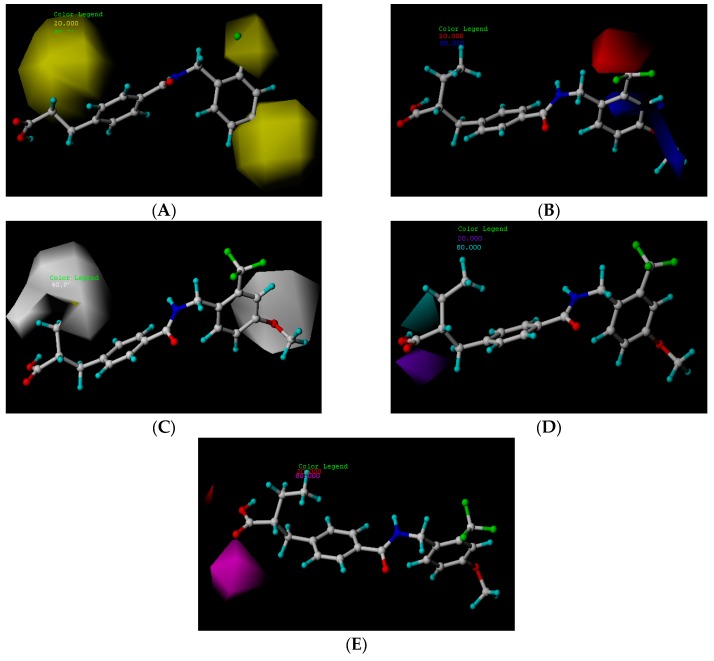
CoMSIA contour maps displayed with most potent compound, **24c**. (**A**) CoMSIA steric contour map (green, favored; yellow, disfavored); (**B**) CoMSIA electrostatic contour map (blue, electropositive favored; red, electronegative favored); (**C**) CoMSIA hydrophobic contour map (yellow, favored; white, disfavored); (**D**) CoMSIA hydrogen donor contour map (cyan, favored; purple, disfavored); (**E**) CoMSIA hydrogen acceptor contour map (magenta, favored; red, disfavored).

The CoMSIA steric and electrostatic contour maps were both similar to the CoMFA contour maps discussed above (Figure 3A,B). Thus, only hydrophobic, hydrogen bond donor, as well as hydrogen bond acceptor fields of CoMSIA, were analyzed in this section. The CoMSIA steric, electrostatic, hydrophobic, hydrogen bond donor along with hydrogen bond acceptor contour maps are shown in Figure 3, respectively. Compound **24c** was selected as the corresponding reference molecule.

#### 2.3.1. Hydrophobic Contour Map

In the hydrophobic contour map (Figure 3C), the yellow contours show favorable hydrophobic regions, while white contours represent unfavorable hydrophobic regions. For the hydrophobic map, one white unfavorable region could be found around the R_2_ substitutes, indicating that the addition of hydrophobic substituents in this region would lead to decrease in activity. Further evidence for this notion can be obtained from compound **30b** bearing a propyl group, which is considerably less hydrophobic than a phenyl group. Therefore, compound **30b** proves to be more active than the biologically less active compound **32b**, bearing a phenyl group. The other white contour area observed in the para position of the benzene ring, indicates that hydrophobic substituents were not preferred in this region. This finding can be further explained by the fact that compound **21c** contains a –Cl substituent that generally leads to a higher potency compared to a methoxy group present in compound **19c**.

#### 2.3.2. Hydrogen Bond Donor Map

The contour map for the hydrogen bond donor field is shown in Figure 3D. Cyan and purple contours represent a hydrogen bond donor field favorable region and hydrogen bond donor unfavorable region, respectively. For the hydrogen bond donor map, a cyan contour appeared around the hydroxyl group. This suggests that a hydrogen bond interaction, with the hydrogen atom of the hydroxyl group acting as a hydrogen bond donor, is favorable for increased activity.

#### 2.3.3. Hydrogen Bond Acceptor Map

In the hydrogen bond acceptor contour map (Figure 3E), the magenta contours represent a favorable hydrogen bond acceptor field, while the red contours represent an unfavorable hydrogen bond acceptor field. For the hydrogen bond acceptor map, one favorable polyhedral surface (magenta) is found around the carboxyl group, which suggests that hydrogen bond interactions between the oxygen atom of the carbonyl group, acting as a hydrogen bond acceptor, and a hydrogen atom of the group, lead to an increase in activity.

### 2.4. Design of More Potent Compounds

Based on CoMFA and CoMSIA models obtained in the present study, the structure–activity relationships of PPARγ agonists could be determined, and several new potent molecules could be designed. The chemical structures of the newly designed compounds, as well as their activity characteristics on PPARγ, were predicted by the CoMFA and CoMSIA models, as seen in Table 2. The predicted activities of the newly designed compounds on PPARγ were all significant. A set of the molecules demonstrated an even better activity than the most active agonist previously reported, further validating the superiority of the models, and indicates that the structure–activity relationships in the work reported herein, may potentially be used in structural modification and optimization.

### 2.5. Docking Analysis

In order to obtain the probable binding conformations between the molecules and the protein, Surflex-dock was carried out to dock the compounds to the binding site of PPARγ. In this study, compound **24c** (template) and a newly designed compound, **N1**, **N9**, and **N12**, were placed in the corresponding binding sites, respectively. The docking score of compound **24c** was 8.913. Meanwhile, the docking scores of compounds **N1**, **N19**, and **N12** were 11.0573, 11.010, and 11.690, respectively. The docking scores of compounds **N1**, **N9**, and **N12** are higher than compound **24c**, which has the highest activity in the training set. This result is in good agreement with corresponding predicted activities of CoMFA and CoMSIA models.

**Figure 4:**
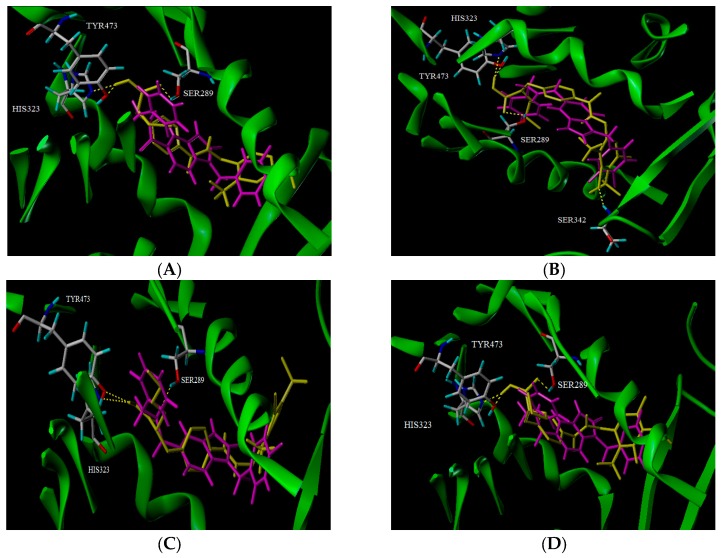

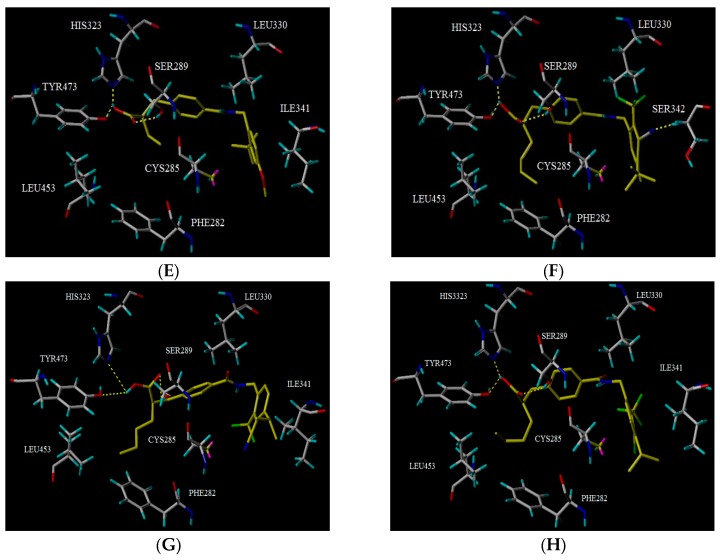
Docking results. (**A**) The surface of the binding site, and the conformation comparison of compound **24c** (yellow), the original ligand (purple), and the key residues (white) at the binding site; (**B**) The surface of the binding site and the comparison of the conformation of, compound **N1** (yellow), the original ligand (purple), and the key residues (white), at the binding site; (**C**) The surface of the binding site, and the comparison of the conformation of compound **N9** (yellow), the original ligand (purple), and the key residues (white), at the binding site; (**D**) The surface of the binding site, and the comparison of the conformation of compound **N12** (yellow), the original ligand (purple), and the key residues (white), at the binding site; (**E**) Interaction between compound **24c** (yellow) and residues (white); (**F**) Interaction between compound **N1** (yellow) and residues (white); (**G**) Interaction between compound **N9** (yellow) and residues (white); (**H**) Interaction between compound **N12** (yellow) and residues (white).

Figure 4 shows the surface of the binding site of PPARγ, the binding modes between compounds **24c**, **N1**, **N9**, and **N12**, and the binding site of the protein. Figure 4A–D, illustrate the surface of the binding site and the conformations of compounds **24c**, **N1**, **N9**, and **N12** (yellow), and the original ligand (purple), as well as the key residues (white) at the binding site. High resemblance between these molecules is observed and they occupied nearly the same binding pocket as PPARγ. It is representative of the active conformation to dock selected compounds. Here, compounds were positioned in the pocket, surrounded by His323, Tyr473, Ser289, Leu453, Ile341, Cys285, etc. As seen in Figure 4E–H, the carbonyl group of the ligand that acts as a hydrogen bond acceptor, formed a hydrogen bond with the backbone O–H of Ser289. The backbone N–H of His323, and O–H of Tyr473, which act as hydrogen bond acceptors and formed hydrogen bonds with the hydroxyl group of the ligand, respectively. Thus, these three hydrogen bond interactions played a major role in the combination of these drugs and the receptor.

## 3. Materials and Methods

### 3.1. Data Set

A set of 33 *N*-benzylbenzamide derivatives and the corresponding activity data were collected from the work of René Blöcher et al. [13] (Table 3). The data set was randomly divided into the training set of 27 compounds (82%) to generate the 3D-QSAR model, and the test set of 6 compounds (18%) to verify the predictive ability of the model. The bioactivities of the compounds were expressed as pEC_50_ (-logEC_50_), which was used as a dependent variable in further investigations. To avoid possible issues during the external validation, the selection of the training and the test set was carried out such that both sets included structurally diverse compounds and all types of activity [16,17,18].

### 3.2. Molecular Modeling and Alignment

**Figure 5:**
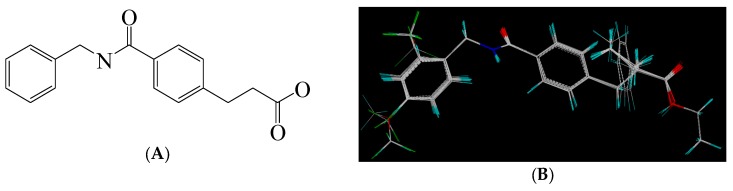
Molecular alignment. (**A**) Common structure retrieved from compound **24**; (**B**) Alignment of the compounds in the training set.

To obtain the best conformers for each molecule, the Sybyl X-2.1.1 software package was used for all compound modeling and optimization parameters. All structures of the compound series were subjected to preliminary geometry optimization using the Tripos force field with 1000 iterations [19]. Partial atomic charges were calculated by the Gasteiger-Hückel scheme, with an energy gradient convergence criterion of 0.05 kcal/mol Å [20]. Based on the analysis method described above, the lowest energy conformation of each molecule was determined for the definitive QSAR studies. Molecular alignment is one of the most essential steps for the generation of the best CoMFA and CoMSIA models [21]. Thus, molecular alignment was performed using the Distill alignment technique, a user-defined common core of the Sybyl tools [22]. Compound **24c**, exhibiting the highest activity in the complete data set, was selected as the template molecule. The remaining compounds in the Mol2 database were aligned by their corresponding maximum common substructures, as shown in Figure 5A. The rigid body alignment of the molecules is shown in Figure 5B.

### 3.3. CoMFA Method

The CoMFA method is often used to describe steric and electrostatic fields. Lennard-Jones and Coulomb potentials were employed to calculate two fields. A 3D cubic lattice, with grid spacing of 2.0 Å, was generated to surround the aligned molecules in all directions. These grid points were generated using the Tripos force field, a sp^3^ carbon atom probe with a Van der Waals radius of 1.52 Å, and a charge of +1.00 (default probe atom in Sybyl). Based on the CoMFA method, steric and electrostatic fields were scaled with a default energy cut off of 30 kcal/mol, the latter being the optimal parameter for this model [23].

### 3.4. CoMSIA Method

The CoMSIA analysis is similar to CoMFA, in regard to the descriptors around the aligned molecules. Three other fields, (i.e., hydrophobic, hydrogen bond donor, and hydrogen bond acceptor fields), were calculated together with the same standard settings used in the CoMFA calculations. More importantly, the distance dependence between the probe atom and each molecule atom was measured by a Gaussian function [24].

### 3.5. Internal Validation and Partial Least Squares (PLS) Analysis

Partial least square (PLS) regression analysis was performed on the training set to construct the correlation between the QSAR model and activity values [25]. To evaluate the reliability of the models generated from PLS analysis, cross-validation analysis was performed through the leave-one-out (LOO) method, which determines the square of the cross-validation coefficient (*q*^2^) and the optimal number of components (ONC). To obtain the non-cross-validation coefficient (*r*^2^), a final non-cross-validation analysis was performed using the ONC derived from cross validation analysis and the corresponding standard error of estimate (SEE). The value for *q*^2^, a measure of the internal quality of the models, was evaluated as follows:

$$q^{2}=\frac{{\sum (y_{obs}-y_{pre})}^{2}}{{\sum (y_{obs}-y_{mean})}^{2}}$$

where *y_obs_*, *y_pre_*, and *y_mean_* are observed, predicted, and mean activity in the training set, respectively.

### 3.6. External Validation of the QSAR Model

To evaluate the predictive ability of CoMFA and CoMSIA models on the test set, the predictive power of the models generated by the CoMFA or CoMSIA analyses with the training set was assessed by calculating the predictive factor *r*^2^ (*r*^2^_pred_) [26], and measuring the predictive performance of the PLS model. The factor *r^2^* was calculated as follows:

$$r_{\mathrm{pread}}^{2}=\frac{\mathrm{SD}-\mathrm{PRESS}}{\mathrm{SD}}$$

the sum of squared deviation, (*SD*) between the biological activity of molecules in the test set and the mean biological activity of the training set molecules; the sum of squared deviations between actual and predicted activity values (*PRESS*), for every molecule in the test set. Coefficients and QSAR results in the contour maps were produced with the field type “STDEV*COEFF”.

### 3.7. Molecular Docking

In an effort to explore the interaction mechanism and investigate suitable binding modes, a molecular docking study was performed using the Sybyl package [27]. The crystal structure of PPARγ was retrieved from the RCSB (Research Collaboratory for Structural Bioinformatics) Protein Data Bank (PDB ID: 5TWO) [28]. In the protein preparation phase, the A-chain was used for the docking study. Crystallized ligands and water molecules of the B-chain were deleted and the hydrogen atoms along with the united atom Gasteiger charges were assigned for the receptor [29]. Based on a protomol generation with a threshold parameter of 0.5 and a bloat parameter of 1 Å, the intended active sites where putative ligands could align to and generate potential interactions, were created using the Sybyl package. Binding affinities were presented by Surflex-Dock total scores. In general, conformations of each ligand were ranked by total scores of docking, with the best conformation of the ligand taken into consideration for the corresponding binding interactions. In this study, compound **24c** (template) and the newly designed compound **N1**, were selected and docked to the binding pocket, using the parameters optimized previously.

## 4. Conclusions

**Figure 6:**
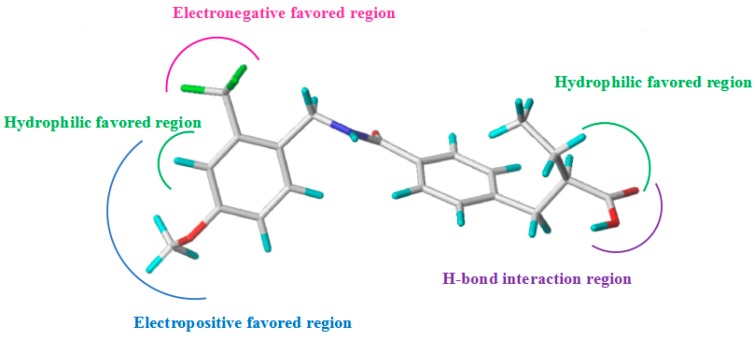
Diagram of structure-activity relationship based on core structure of template compound **24c**.

In this paper, 3D-QSAR and molecular docking studies were utilized to investigate the structural requirements for improving the potency of *N*-benzylbenzamide derivatives as PPARγ agonists. The established CoMFA and CoMSIA models were both statistically significant, with high external prediction characteristics, indicating that the models could be used to successfully predict compound activity. Surflex-Dock analysis also demonstrated the binding interactions of the template compound with amino acids. Using the model parameter analysis and contour maps, the corresponding structure-activity relationships were determined (Figure 6). Based on the information derived from the different contour maps, several new compounds with improved activities, were designed, further validating the ability of the generated model. We surmise that this will be helpful for the future development of new PPARγ agonists, in the design and screening of new high-activity compounds.

## Figures and Tables

**Table 1:** Statistical parameters of the CoMFA and CoMSIA models.

PLS Statistics	ONC	*q*^2^	*r*^2^	SEE	F	Cotribution (%)				
						S	E	H	D	A
*CoMFA*										
S	8	0.576	0.930	0.132	29.872	100	–	–	–	–
E	7	0.497	0.937	0.122	40.470	–	100	–	–	–
SE	6	0.750	0.958	0.097	76.113	51.6	48.4	–	–	–
*CoMSIA*										
S	5	0.472	0.804	0.205	17.183	100	–	–	–	–
E	7	0.428	0.911	0.145	27.952	–	100	–	–	–
H	10	0.506	0.958	1.108	36.771	–	–	100	–	–
D	2	−0.051	0.168	0.395	2.418	–	–	–	100	–
A	1	−0.083	0.030	0.418	0.777	–	–	–	–	100
SE	10	0.61	0.949	0.120	29.708	34.0	66.0	–	–	–
SH	3	0.412	0.823	0.186	35.545	36.1	–	63.9	–	–
SD	10	0.505	0.935	0.135	23.071	48.3	–	–	51.7	–
SA	4	0.493	0.803	0.201	22.415	84.9	–	–	–	15.1
EH	5	0.479	0.891	0.153	34.255	–	59.1	40.9	–	–
ED	9	0.352	0.932	0.134	25.959	–	88.1	–	11.9	–
EA	6	0.433	0.876	0.167	23.640	–	89.1	–	–	10.9
HD	10	0.537	0.965	0.100	43.744	–	–	75.1	24.9	–
HA	10	0.525	0.958	0.109	36.225	–	–	90.6	–	9.4
DA	2	−0.036	0.186	0.391	2.740	–	–	–	81.0	19.0
SEH	5	0.541	0.916	0.134	45.732	17.9	51.9	30.2	–	–
SED	10	0.6	0.954	0.113	33.508	32.0	55.9	–	12.1	–
SEA	9	0.607	0.944	0.121	32.005	34.9	60.4	–	–	4.7
SHD	5	0.448	0.913	0.137	43.915	28.9	–	51.0	20.1	–
SHA	5	0.426	0.909	0.140	41.769	33.8	–	59.6	–	6.6
SDA	4	0.501	0.800	0.202	22.018	57.8	–	–	32.2	9.9
EHD	5	0.478	0.885	0.157	32.229	53.1	–	35.9	11.0	–
EHA	5	0.492	0.889	0.154	33.757	–	56.8	38.9	–	4.3
EDA	9	0.368	0.941	0.125	30.157	–	79.6	–	11.8	8.6
HDA	10	0.532	0.964	0.100	43.071	–	–	70.1	23.5	6.4
SEHD	5	0.545	0.912	0.137	43.732	16.1	47.7	27.1	9.1	–
SEHA	5	0.55	0.915	0.135	45.170	17.3	50.5	28.9	–	3.3
SEDA	10	0.593	0.954	0.113	33.520	30.3	52.9	–	11.7	5.1
SHDA	5	0.449	0.905	0.143	39.940	28.1	–	49.5	17.5	4.9
EHDA	5	0.486	0.884	0.159	32.044	–	51.6	35.0	9.7	3.7
SEHDA	5	0.551	0.912	0.138	43.388	15.7	46.7	26.5	8.2	2.8

Optimum number of components (ONC), leave-one-out cross-validated correlation coefficient (*q*^2^), noncross-validated correlation coefficient (*r*^2^), standard error of estimate (SEE), Fischer test values (F), steric field (S), electrostatic field (E), hydrophobic field (H), Hydrogen bond donor field (D), Hydrogen bond acceptor field (A).

**Table 2:**
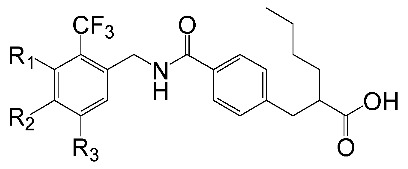
Newly designed compounds and predictive activity.

NO.	R_1_	R_2_	R_3_	CoMFA Predicted	CoMSIA Predicted
N1	CN	C(Me)_3_	Me	6.942	7.170
N2	CN	C(Me)_3_	OMe	6.765	7.155
N3	CN	C(Me)_3_	ET	6.900	7.155
N4	CN	CH(Me)_2_	OMe	6.982	7.105
N5	CN	C(Me)_3_	–	6.822	7.083
N6	CN	CH(Me)_2_	Me	7.064	7.080
N7	CN	CH(Me)_2_	ET	7.103	7.070
N8	COOH	C(Me)_3_	Me	6.820	7.063
N9	CN	CH(Me)_2_	–	7.036	6.999
N10	Cl	C(Me)_3_	Me	6.742	6.986
N11	CHO	C(Me)_3_	Me	6.878	6.916
N12	COOH	CH(Me)_2_	Me	7.073	6.902
N13	CHO	C(Me)_3_	c-Pr	6.815	6.886
N14	Cl	CH(Me)_2_	Me	6.781	6.843
N15	–	C(Me)_3_	–	6.648	6.798

**Table 3:**
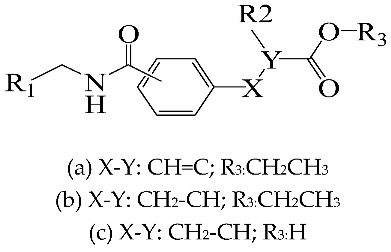
Actual and predicted pEC_50_ values of PPARγ agonists.

NO.	R_1_	R_2_	Substitution	Actual pEC_50_	Pred-pEC_50_	
					CoMFA	CoMSIA
01a *	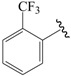	Et	para	5.000	4.996	4.992
02b	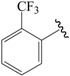	Et	para	5.745	5.650	5.655
03c *	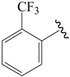	Et	para	5.319	5.483	5.420
04b	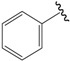	Et	para	4.796	4.915	4.867
05c	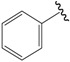	Et	para	4.870	4.869	4.911
06b	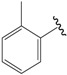	Et	para	5.000	4.941	4.845
07b	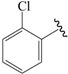	Et	para	5.000	5.015	5.029
08c	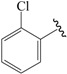	Et	para	5.000	5.039	5.093
09c	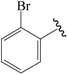	Et	para	5.000	5.019	5.053
10b *	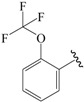	Et	para	5.456	5.174	5.317
11c	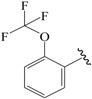	Et	para	5.097	5.090	5.097
12b	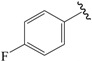	Et	para	4.959	4.874	5.028
13c	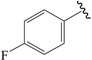	Et	para	5.000	4.924	5.125
14b	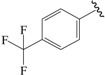	Et	para	5.377	5.261	5.178
15c	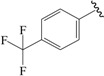	Et	para	5.201	5.315	5.297
16b	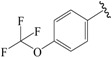	Et	para	5.523	5.588	5.607
17c	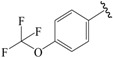	Et	para	5.699	5.645	5.712
18b	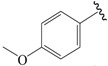	Et	para	5.000	5.060	4.984
19c	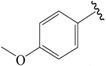	Et	para	5.155	5.121	5.089
20b	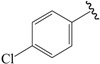	Et	para	5.000	5.182	5.047
21c	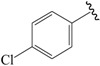	Et	para	5.398	5.239	5.156
22b *	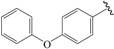	Et	para	5.854	5.846	5.551
23b	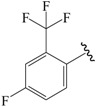	Et	para	5.553	5.615	5.841
24c	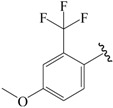	Et	para	6.523	6.542	6.349
25b *	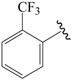	Et	meta	5.000	5.052	4.991
26c *	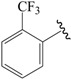	Et	meta	5.194	5.008	4.983
27b	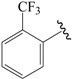	H	para	5.000	4.966	4.934
28c	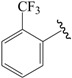	H	para	5.000	5.002	4.974
29b	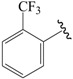	Me	para	5.097	5.151	5.245
30b	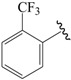	Pr	para	6.046	5.875	5.886
31c	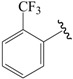	Pr	para	5.824	5.933	5.993
32b	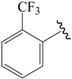	Phenyl	para	5.699	5.699	5.648
33c	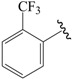	Phenyl	para	5.523	5.556	5.439

⁎ Test set molecules.
